# Genetic variation in *NFE2L2* is associated with outcome following aneurysmal subarachnoid haemorrhage

**DOI:** 10.1111/ene.15571

**Published:** 2022-10-02

**Authors:** Ben Gaastra, Poppy Duncan, Mark K. Bakker, Isabel C. Hostettler, Varinder S. Alg, Henry Houlden, Ynte M. Ruigrok, Ian Galea, Will Tapper, David Werring, Diederik Bulters

**Affiliations:** ^1^ Clinical Neurosciences, Clinical & Experimental Sciences, Faculty of Medicine University of Southampton Southampton UK; ^2^ Department of Neurosurgery, Wessex Neurological Centre University Hospital Southampton Southampton UK; ^3^ Department of Neurology University Medical Center Utrecht Brain Center, University Medical Center Utrecht Utrecht the Netherlands; ^4^ Stroke Research Centre University College London, Institute of Neurology London UK; ^5^ Department of Neurosurgery Kantonsspital St. Gallen St. Gallen Switzerland

**Keywords:** NF‐E2‐related factor 2, polymorphism, single nucleotide, subarachnoid haemorrhage

## Abstract

**Background and purpose:**

Nuclear factor erythroid 2‐related factor 2 (NRF2; encoded by the *NFE2L2* gene) has been implicated in outcome following aneurysmal subarachnoid haemorrhage (aSAH) through its activity as a regulator of inflammation, oxidative injury and blood breakdown product clearance. The aim of this study was to identify whether genetic variation in *NFE2L2* is associated with clinical outcome following aSAH.

**Methods:**

Ten tagging single nucleotide polymorphisms (SNPs) in *NFE2L2* were genotyped and tested for association with dichotomized clinical outcome, assessed by the modified Rankin scale, in both a discovery and a validation cohort. *In silico* functional analysis was performed using a range of bioinformatic tools.

**Results:**

One SNP, rs10183914, was significantly associated with outcome following aSAH in both the discovery (*n* = 1007) and validation cohorts (*n* = 466). The risk of poor outcome was estimated to be 1.33‐fold (95% confidence interval 1.12–1.58) higher in individuals with the T allele of rs10183914 (*p*_meta‐analysis_ = 0.001). *In silico* functional analysis identified rs10183914 as a potentially regulatory variant with effects on transcription factor binding in addition to alternative splicing with the T allele, associated with a significant reduction in the *NFE2L2* intron excision ratio (*p*_sQTL_ = 1.3 × 10^−7^).

**Conclusions:**

The *NFE2L2* SNP, rs10183914, is significantly associated with outcome following aSAH. This is consistent with a clinically relevant pathophysiological role for oxidative and inflammatory brain injury due to blood and its breakdown products in aSAH. Furthermore, our findings support NRF2 as a potential therapeutic target following aSAH and other forms of intracranial haemorrhage.

## INTRODUCTION

Aneurysmal subarachnoid haemorrhage (aSAH) is a devastating form of stroke associated with significant morbidity and mortality [1]. It has worse outcomes and occurs in younger people compared with other forms of stroke, meaning aSAH results in the greatest socioeconomic burden of all stroke types [2].

The initial surge in intracranial pressure caused by aneurysm rupture instigates early brain injury [3]. The toxic cascades initiated in this early brain injury, together with blood and its breakdown products, which are gradually released into the cerebrospinal fluid (CSF) as the clot lyses, lead to delayed brain injury characterized by cerebral vasospasm, inflammation, oxidative injury and cortical spreading depression [4, 5, 6, 7]. Oxidative injury is thought to play a key role in the pathophysiology of neurological injury following aSAH [8]. There is significant evidence of oxidative stress within human CSF following aSAH, with a higher oxidative burden associated with complications and worse outcomes [9, 10, 11].

Nuclear factor erythroid 2‐related factor 2 (NRF2) has been demonstrated in humans and animals to play an important role in oxidative and neurological injury following aSAH. NRF2, encoded by the *NFE2L2* gene in humans, is a transcription factor which responds to oxidative stress by upregulating multiple antioxidants including glutathione S‐transferase and peroxiredoxin [12]. It also regulates transcription of blood breakdown product scavenging molecules such as heme oxygenase‐1 (HO‐1) [12] and haptoglobin (Hp) [13]. NRF2 has also been shown to have anti‐inflammatory properties [14]. It is activated following aSAH in response to oxidative stress and by blood breakdown products released into the CSF following haemorrhage [5]. Consequently, NRF2 plays a potentially pivotal role in protection from neurological injury and outcome following aSAH, not only by upregulating antioxidants and dampening inflammation but also by promoting the clearance of blood breakdown products which drive the oxidative and inflammatory response to aSAH [15, 16].

Nrf2 has been implicated in the pathophysiology of aSAH in multiple animal studies. Nrf2 is upregulated in the cerebral vasculature and cortex following experimental subarachnoid haemorrhage (SAH) in rodents [17, 18]. Nrf2 knockout mice exposed to experimental SAH have increased brain injury and neurological deficits compared to wild‐type mice [19]. Numerous studies have demonstrated that pharmacological upregulation of Nrf2 is associated with reduced oxidative and neurological injury, with improved outcomes in rodent SAH models [20, 21]. Sulforaphane is a pharmacological upregulator of Nrf2 activity, which in rodent SAH models reduces neurological injury, cerebral vasospasm and inflammatory cytokines [18, 22].

*NFE2L2* is a highly polymorphic gene for which genetic variation has been associated with human disease risk including in respiratory, gastrointestinal and haematological conditions [23]. In neurological disease, genetic variation in *NFE2L2* has been associated with Parkinson's disease and amyotrophic lateral sclerosis incidence and age of onset [24, 25]. In the context of human aSAH, genetic variation in *NFE2L2* has not been specifically studied.

Given its pivotal role in both oxidative and inflammatory injury and its association with the pathophysiology of a wide spectrum of diseases, we hypothesized that genetic variation in *NFE2L2* influences outcome following aSAH. The aims of this study were to investigate whether genetic variation within *NFE2L2* is associated with outcome following aSAH and to validate the findings in an external cohort.

## METHODS

This was an *NFE2L2* candidate gene study to test for an association between single nucleotide polymorphisms (SNPs) and outcome following aSAH in a discovery and a validation cohort.

This study had both national (REC 19/SC/0485) and local (ERGO 49253) ethical approval. Patients, or next of kin if patients lacked capacity, gave written informed consent. The study is reported according to the STREGA recommendations [26].

### Discovery analysis

#### Subjects

DNA and phenotype information were obtained from patients with aSAH recruited to the Genetics and Observational Subarachnoid Haemorrhage (GOSH) study. The GOSH study recruited patients from 22 neurosurgical centres in the United Kingdom between 2011 and 2014, and was designed to study the genetic characteristics of aSAH, the details of which have previously been reported [27]. All individuals with available DNA, confirmed aSAH and suitable data on clinical outcome were eligible for inclusion in this study.

#### Outcomes and covariates

The primary outcome was the dichotomized modified Rankin scale (mRS) score at follow‐up. An mRS score of 0–1 was defined as good outcome and an mRS score of 2–6 as poor outcome. This high threshold for good outcome was prespecified in view of the known long follow‐up periods (up to 8 years) and consequently good outcomes in this cohort [28, 29]. The following prespecified covariates were included in the analysis: World Federation of Neurological Surgeons (WFNS) grade; Fisher grade [30]; treatment (conservative, endovascular, surgical); time to follow‐up; sex; and age. All covariates were tested as categorical apart from age and time to follow‐up, which were treated as continuous variables. Multilevel categorical data were converted to a set of binary dummy variables for analysis. Missing covariate data were imputed using a method of polytomous regression for multilevel categorical data and predictive mean matching for continuous data [31].

#### SNP selection and genotyping

Tagging SNPs within and surrounding the *NFE2L2* gene were identified using a linkage‐disequilibrium‐based mapping approach [25] to efficiently capture as much common variation as possible. Eight tagging SNPs were identified from HapMap data (release 28) [32] including regions 5 kb downstream to 10 kb upstream of *NFE2L2* (minor allele frequency >5%, *r* ^2^ = 0.9). A further two tagging SNPs were identified from the upstream regulatory region (see Table 1 for details of tagging SNPs). These SNPs were genotyped by Kompetitive Allele‐Specific PCR (KASP) at LGC Genomics.

**TABLE 1:** Tagging single nucleotide polymorphisms included in analysis

Chromosome	SNP	Base pair	Location	Major allele	Minor allele	Minor allele frequency
2	rs13035806	177,227,094	Intergenic	G	A	0.18
2	rs2706110	177,227,434	Intergenic	C	T	0.20
2	rs10183914	177,232,938	Intronic	C	T	0.34
2	rs6726395	177,238,501	Intronic	G	A	0.45
2	rs10930781	177,249,904	Intronic	G	A	0.09
2	rs1806649	177,253,424	Intronic	C	T	0.23
2	rs2364723	177,261,818	Intronic	G	C	0.31
2	rs6706649	177,265,343	Upstream	C	T	0.13
2	rs35652124	177,265,345	Upstream	T	C	0.31
2	rs6433657	177,269,949	Intergenic	G	A	0.45

*Note*: Base pair is reported in reference to hg38. Minor allele frequency is reported for the Genetics and Observational Subarachnoid Haemorrhage (GOSH) study cohort.

Abbreviation: SNP, single nucleotide polymorphism.

#### Quality control

Candidate SNPs were excluded if the genotyping rate was <90% for the cohort, minor allele frequency was <0.05 or there was significant deviation from Hardy–Weinberg equilibrium (*p* < 0.0001).

#### Association analysis

Individual SNPs were tested for association with dichotomized outcome under an additive model with logistic regression controlling for all covariates. Haplotypes including all 10 SNPs and a frequency ≥0.01 were tested for association using the same logistic regression model. As this was an exploratory analysis, correction for multiple testing was not performed.

### Validation analysis

The SNPs and haplotypes associated with outcome (*p* < 0.05) were assessed for replication in an independent cohort of Dutch aSAH patients who were recruited from the University Medical Centre Utrecht, The Netherlands. mRS scores for all individuals were determined at 3 months after aSAH. DNA was extracted from blood and genotyped on either Illumina GSA or CNV370‐duo platforms. Additional SNPs were imputed on the Michigan Imputation Server. Further details about the genotyping and their quality control have been published elsewhere [33]. Array genotype data underwent quality control including exclusion of individuals with >10% missingness; SNPs were excluded if minor allele frequency was <0.05, there was extreme deviation from the Hardy–Weinberg equilibrium (*p* < 0.0001) or the SNP call rate was <90%.

In the validation cohort an mRS score of 0–2 was defined as good outcome and an mRS score of 3–6 as poor outcome. This was a different threshold from that used in the discovery cohort in view of the fact that follow‐up in this cohort was at 3 months. This is a more common assessment time in aSAH studies and, consequently, the threshold for good outcome most often employed in aSAH studies was applied. This approach ensured comparable numbers of patients with poor outcome in the discovery and validation cohorts for statistical analysis. To explore the effect of these mRS score thresholds, the primary analysis was repeated using the same threshold as that used in the validation cohort (see sensitivity analysis).

The same logistic regression model was used to validate the association between significant SNPs and haplotypes with outcome following aSAH. Age, sex and WFNS grade were included as covariates in the model. All individuals were followed up at the same time point and, consequently, follow‐up time was not required as a covariate in the model. Fisher grade and treatment status were not available for this dataset. The Bonferroni method for multiple corrections was applied based on the number of SNPs and haplotypes tested in the replication cohort.

The final effect size for statistically significant validated SNPs was identified by a fixed effects meta‐analysis of summary statistics from the discovery and validation cohorts.

#### Functional analysis

Genetic variants which replicated in the validation cohort underwent *in silico* functional analysis to assess their biological relevance. The RegulomeDB probability score was used to assess the likelihood of any variant being regulatory [34]. UCSC Genome Browser [35, 36, 37] and HaploReg (version 4.1) [38] were used to annotate against the 15‐state chromatin model [39] to identify whether relevant variants and their proxies (*r* ^2^ > 0.85) had significant chromatin interactions. GTEx (version 8) [40] was used to assess whether variants or their proxies (*r* ^2^ > 0.85) were associated with gene expression levels (eQTL) and/or alternative splicing (sQTL).

#### Sensitivity analyses

The primary analysis was also repeated in the GOSH cohort using mRS score 0–2 to define good outcome, the same dichotomization threshold as used in the validation cohort. As Fisher grade and treatment status were not available in the validation cohort, the primary analysis was repeated in the GOSH cohort excluding these covariates.

All analyses were performed in STATA (StataCorp. 2011. Stata Statistical Software: Release 16. College Station, TX: StataCorp LP) and PLINK versions 1.07 and 1.9.

#### Data availability

Study data will be available from the authors subject to institutional agreements and ethical approvals.

## RESULTS

### Discovery cohort

**FIGURE 1:**
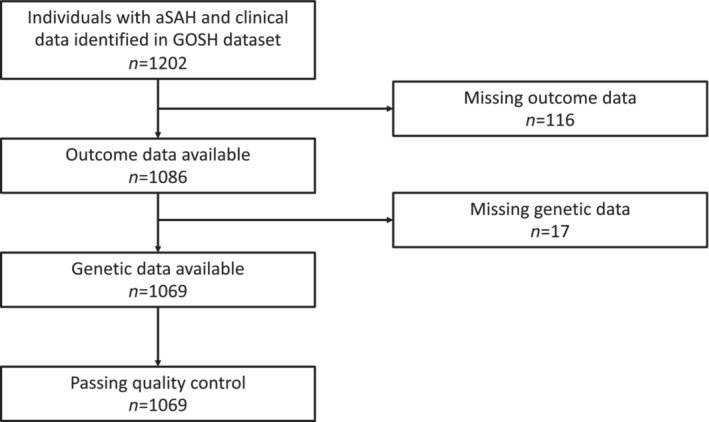
Flow chart for Genetics and Observational Subarachnoid Haemorrhage (GOSH) study sample inclusion. aSAH, aneurysmal subarachnoid haemorrhage

A total of 1202 patients with aSAH were identified from the GOSH dataset, of whom 116 individuals were excluded due to missing outcome data. Of the remaining 1086 samples, 17 (1.6%), 57 (5.2%), 10 (0.9%) and 18 (1.7%) were missing WFNS grade, Fisher grade, treatment status and time to follow‐up, respectively, and were imputed. Genetic data were available on 1078 patients, of whom 1069 passed quality control thresholds. After dichotomization on mRS score, 68% of patients (*n* = 732) were defined as having a good outcome (mRS score 0–1) and 32% (*n* = 337) had a poor outcome (mRS score 2–6). The mean (range) follow‐up time was 24.7 (0–96) months (Figure S1). See Figure 1 for flow chart of sample inclusion and Table 2 for demographics of included patients.

**TABLE 2:** Demographics of patients included from discovery and validation cohort

Cohort	Discovery: GOSH cohort	Validation: Utrecht cohort
Sample size	1069	466
Mean (range) age, years	53.6 (19–92)	57.3 (23–92)
WFNS grade, *n* (%)		
1	602 (56)	216 (46)
2	237 (22)	126 (27)
3	53 (5)	23 (5)
4	104 (10)	57 (12)
5	73 (7)	44 (9)
Fisher grade, *n* (%)		
1	83 (8)	Not available
2	321 (30)	
3	241 (23)	
4	424 (40)	
Mean (range) time to follow‐up, months	24.7 (0–96)	3
Treatment, *n* (%)		
Conservative	9 (1)	Not available
Endovascular	846 (79)	
Surgical	214 (20)	
Sex, *n* (%)		
Female	754 (71)	339 (73)
Male	315 (29)	127 (27)
Outcome, *n* (%)		
mRS score 0	354 (33)	5 (1)
mRS score 1	378 (35)	25 (5)
mRS score 2	158 (15)	318 (68)
mRS score 3	85 (8)	19 (4)
mRS score 4	36 (3)	32 (7)
mRS score 5	15 (1)	30 (6)
mRS score 6	43 (4)	37 (8)
Good outcome^a^	732 (68)	348 (75)
Poor outcome	337 (32)	118 (25)

*Note*: Good outcome was defined as mRS score 0–1 and mRS score 0–2 in the discovery and validation cohorts, respectively. Discovery demographics are reported following imputation.

Abbreviations: GOSH, Genetics and Observational Subarachnoid Haemorrhage; mRS, modified Rankin scale; WFNS, World Federation of Neurological Surgeons.

### Association analysis

Multivariable logistic regression identified two candidate SNPs significantly associated with outcome following aSAH and adjusting for known prognostically relevant covariates (age, Fisher grade, time to follow‐up, sex, treatment and WFNS grade): the rs10183914 T allele was associated with poor outcome with an odds ratio (OR) of 1.27 (95% confidence interval [CI] 1.04–1.55; *p* = 0.021, *n* = 1007) and the rs6433657 A allele was associated with poor outcome with an OR of 1.24 (95% CI 1.02–1.50; *p* = 0.034, *n* = 1002 [Table 3 and Tables S1–S3]). Two haplotypes were associated with outcome using the same multivariable analysis to adjust for covariates (Table S4). Of these two haplotypes the haplotype containing the rs10183914 T and rs6433657 A risk alleles was associated with an increased risk of poor outcome (OR 1.39), whereas the haplotype containing the rs10183914 C and rs6433657 G alleles was protective, with a reduced risk of poor outcome (OR 0.59).

**TABLE 3:** Multivariable logistic regression of significant single nucleotide polymorphisms for the Genetics and Observational Subarachnoid Haemorrhage (GOSH) and Utrecht study cohorts.

			GOSH cohort			Utrecht cohort		
SNP	Base pair	Minor allele	Number of samples	OR (95% CI)	*p* value	Number of samples	OR (95% CI)	*p* value
rs10183914	177,232,938	T	1007	1.27 (1.04–1.55)	0.021^a^	466	1.5 (1.07–2.10)	0.019^b^
rs6433657	177,269,949	A	1002	1.24 (1.02–1.50)	0.034^a^	466	1.07 (0.77–1.48)	0.693

*Note*: ORs are reported with respect to the minor allele. Base pair reported in reference to hg38. Genotypes for rs10183914 in the GOSH cohort: CC 451, CT 418, TT 138; Utrecht cohort: CC 177, CT 211, TT 78; for rs6433657 in the GOSH cohort: GG 327, AG 455, AA 220; Utrecht cohort: GG 121, AG 223, AA 122.

Abbreviations: OR, odds ratio; SNP, single‐nucleotide polymorphism.

^a^ Signifies <0.05. In the validation cohort.

^b^ Represents significance following Bonferroni correction (<0.025).

#### Validation analysis

A total of 466 patients were identified from the University Medical Centre Utrecht dataset, all of whom passed quality control parameters (see Table 2 for demographics). In all, 348 patients (75%) had a good outcome (mRS score 0–2) and 118 (25%) a poor outcome (mRS score 3–6).

**FIGURE 2:**
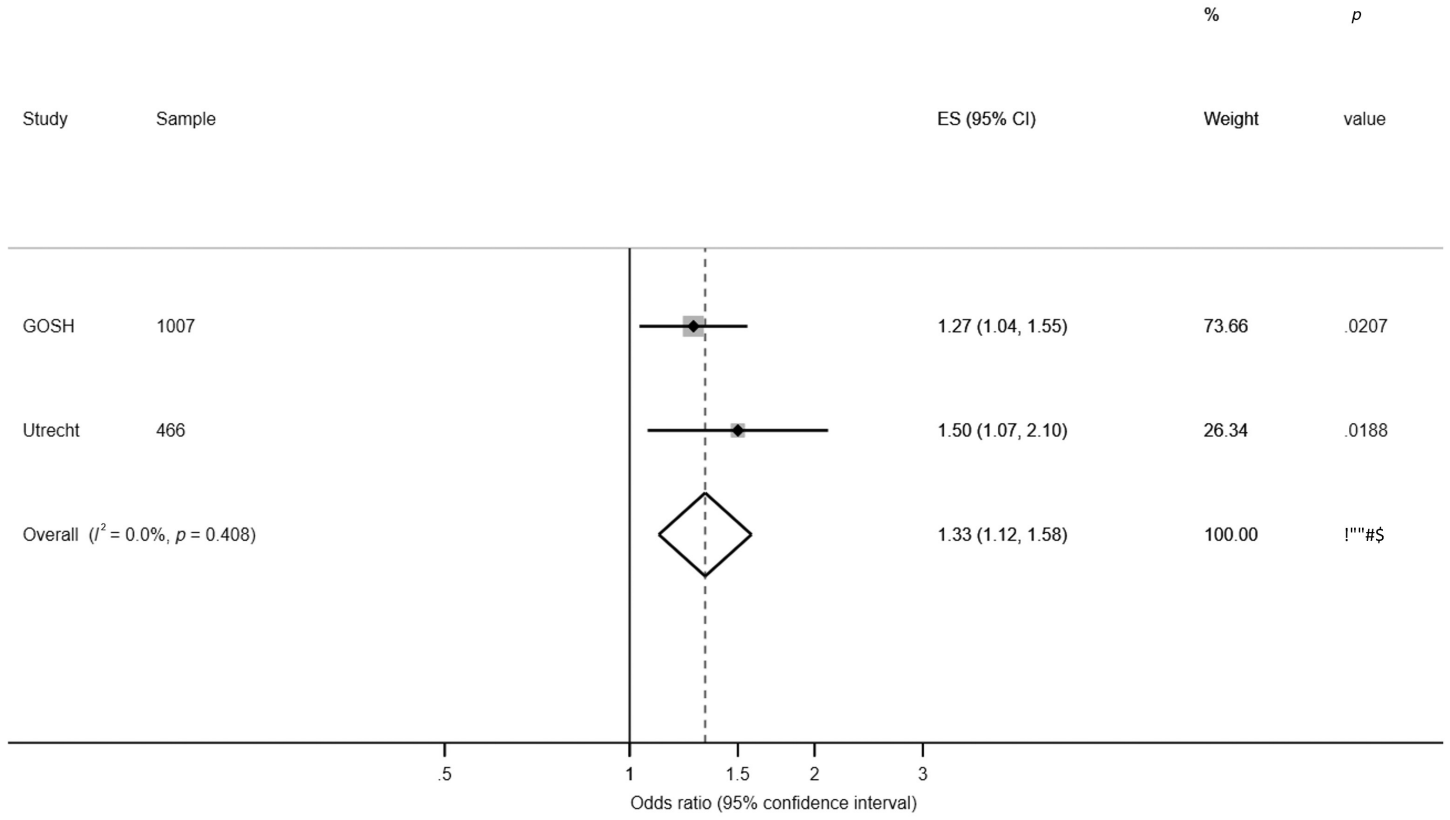
Forest plot displaying summary results for rs10183914 in the Genetics and Observational Subarachnoid Haemorrhage (GOSH) study (discovery) and Utrecht (validation) cohorts

The two statistically significant associated SNPs (rs10183914 and rs6433657) were tested for replication in the Utrecht cohort using a logistic regression model controlling for age, sex and WFNS grade. Using Bonferroni correction (corrected *p* value <0.025), the rs10183914 T allele replicated with a statistically significant association with outcome in the Utrecht cohort (*p* = 0.019). The rs6433657 A allele did not replicate in the Utrecht cohort (*p* = 0.693) (Table 3). In a fixed‐effects meta‐analysis of the discovery and validation cohorts the odds of a poor outcome were estimated to be 1.33 times higher per copy of the risk T allele (95% CI 1.12–1.58; *p* = 0.001), with no evidence of heterogeneity (*I* ^2^ = 0%; Figure 2).

The two significant haplotypes were also tested for validation in the Utrecht cohort using the same multivariable logistic regression model. Neither of the haplotypes replicated in the Utrecht dataset.

### Functional analysis

**FIGURE 3:**
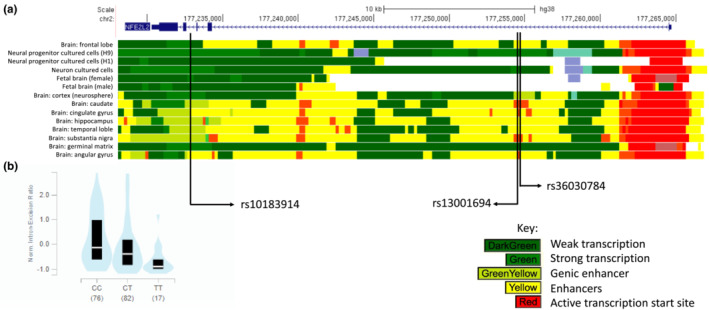
*In silico* functional analysis showing relevant SNPs. (a) 15‐state chromatin model in relevant tissue (http://genome.ucsc.edu). (b) *NFE2L2* splicing QTL analysis for rs10183914 (http://gtexportal.org).

rs10183914 has a RegulomeDB probability score of 0.609, suggesting this is a putative regulatory variant with evidence of transcription and enhancer activity in brain tissue. Interrogation of HaploReg epigenomic information highlighted a cluster of enhancers, defined by the 15‐state chromatin model [39], within the brain, associated with two SNPs (rs13001694 and rs36030784) in strong linkage disequilibrium with rs10183914 (*r* ^2^ > 0.85, *D*′ = 0.95; Figure 3). HaploReg and RegulomeDB also identified that rs10183914 alters regulatory motifs for transcription factors Foxc1, CEBPB and PBX2. rs10183914 is also associated with significant splicing quantitative trait loci changes in brain cortex, with the T allele associated with a significantly reduced *NFE2L2* intron excision ratio (*p* = 1.3 × 10^−7^) as analysed by LeafCutter [41] (Figure 3). Finally, rs10183914 is associated with significantly altered expression of *NFE2L2* using eQTL analysis, for example, in cultured fibroblasts, although not in brain tissue [40].

### Sensitivity analyses

When an mRS score of 0–2 was used to define good outcome in the GOSH study cohort, only 17% of individuals were classified as having poor outcome. Using this definition the rs10183914 T allele had an OR of poor outcome of 1.27 (95% CI 0.98–1.62; *p* = 0.076, *n* = 1007) and the rs6433657 A allele had an OR of poor outcome of 1.24 (95% CI 0.85–1.40; *p* = 0.480, *n* = 1002). A fixed‐effects meta‐analysis of rs10183914 in the discovery and validation cohorts identified a highly significant estimated OR of 1.34 (95% CI 1.09–1.64; *p* = 0.005). Exclusion of Fisher grade and treatment status as covariates had minimal effect on the significance of the relationship between genotype and outcome in the GOSH study cohort (excluding Fisher grade: rs10183914, *p* = 0.029; rs6433657, *p* = 0.046; excluding treatment status: rs10183914, *p* = 0.017; rs6433657, *p* = 0.043).

## DISCUSSION

In this *NFE2L2* candidate gene study we identified and externally validated the novel finding that the rs10183914 T allele is associated with poor clinical outcome following aSAH in humans (OR 1.33; 95% CI 1.12–1.58).

NRF2 has been proposed to play an integral role in neurological injury and outcome following aSAH by promoting clearance of blood and its breakdown products and protecting against oxidative and inflammatory injury [15, 16]. At present, however, there is only rodent data supporting a role for Nrf2 in SAH [19, 20]. By identifying genetic variation within *NFE2L2* associated with outcome, this study demonstrates the relevance of NRF2 to functional outcome in humans following aSAH. This is consistent with a clinically relevant pathophysiological role for oxidative and inflammatory brain injury due to blood and its breakdown products following aSAH in humans. It also highlights NRF2 as a strong potential therapeutic target to mitigate the devastating consequences of this condition. At present there is an ongoing clinical trial to assess the impact of sulforaphane, a NRF2 stabilizer, in aSAH [42].

In this study the presence of the rs10183914 T allele was associated with poor outcome following aSAH (OR 1.33; 95% CI 1.12–1.58). The functional effect of the intronic rs10183914 is, however, unknown. The alternate T allele has been associated with later age of onset in Parkinson's disease, although the effect of the genetic variant is unknown, with no change in expression of *NFE2L2* in human olfactory neurosphere‐derived cell lines [25]. *In silico* functional analysis links both rs10183914 and SNPs in strong linkage disequilibrium, rs13001694 and rs36030784, to gene regulation, suggesting a significant functional role for this variant. Splicing QTL analysis identified that rs10183914 significantly alters the intron‐excision ratio of NFE2L2 specifically in brain tissue (*p* = 1.3 × 10^−7^), not only supporting a functional effect of the variant but also highlighting it specifically in neural tissue. Splicing significantly contributes to transcriptome and subsequent proteome diversity [43] and the risk rs10183914 T allele is associated with a reduced intron‐excision ratio, leading to lower transcription diversity in the brain and potentially a longer protein, which is likely to be dysfunctional. rs10183914 also significantly influences expression of NRF2 although not specifically in brain tissue. Further *in vitro* analysis is required to characterize the functional effect of this variant and inform future studies.

The findings of this study also have implications for other haemorrhagic stroke conditions such as intracerebral haemorrhage, where Nrf2 activity has also been shown to be neuroprotective in rodent models [44]. As genetic variation influences outcome after aSAH in humans this may translate to other conditions, such as intracerebral haemorrhage, where NRF2 may also be a promising therapeutic target to improve outcome, building on the body of animal evidence available [45].

In this study, only 10 tagging SNPs were genotyped, all of which are common, with a minor allele frequency > 0.05. Despite this small number of variants, a significant association with outcome following aSAH was identified. Future studies including a wider range of common and rare genetic variants may identify stronger relationships to outcome, further supporting an integral role for *NFE2L2* in aSAH recovery.

This finding adds to the growing body of evidence that genetic variation plays an important role in outcome following aSAH [46, 47] and supports the ongoing effort to perform a genome‐wide association study of outcome following aSAH [48].

A strength of this study is the long‐term follow‐up duration in the GOSH cohort, ranging from 0 to 96 months. This suggests that the effect of NRF2 on outcome is not simply an acute phenomenon but persists in the long term. The wide range of follow‐up in the GOSH dataset is complemented by the fixed shorter‐term follow‐up time in the Utrecht dataset (3 months), both of which identify a significant association between rs10183914 and outcome, supporting the significance of this variant in both the short and long term.

The difference in follow‐up duration between the two cohorts, however, results in a higher proportion of individuals with mRS score 0–1 in the GOSH study cohort (Table 2) due to longer recovery time before outcome assessment. For this reason, we prespecified different mRS dichotomization thresholds for good versus poor outcome in the discovery (GOSH; mRS 0–1 vs. 2–6), and validation (Utrecht; mRS 0–2 vs. 3–6) cohorts. Both these dichotomization thresholds are used in the literature in the context of outcome following aSAH [27, 49]. An mRS score of 0–2 is more commonly used to define good outcome in studies with follow‐up at 3–6 months (as in the Utrecht cohort), whereas the mRS score 0–1 dichotomy has been applied to longer‐term follow‐up cohorts such as that of the GOSH study. The differing dichotomies were used in this study to ensure equivalent proportions of good/poor outcome individuals in the two cohorts and to allow robust statistical analysis (Table 2). A sensitivity analysis was performed using mRS scores of 0–2 to define good outcome in the GOSH study cohort and showed the same direction of effect (OR 1.24 compared to 1.27), with a suggestive *p* value of 0.076, and meta‐analysis of the discovery and validation cohorts remained highly significant (OR 1.34, 95% CI 1.09–1.64; *p* = 0.005), supporting the findings of the primary analysis.

Fisher grade and treatment status were missing in the Utrecht cohort, preventing their inclusion in the multivariable model for the validation cohort. However, this is very unlikely to have influenced the significance of the results. Firstly, Fisher grade and treatment status have minimal influence on outcome, explaining only 0.65% and 1.3% of outcome variation, respectively, in the largest predictive modelling study to date [50] and 4.6% and 0.4% variation in outcome, respectively, in the GOSH dataset. Secondly, exclusion of Fisher grade and treatment status from the GOSH analysis only had a minimal impact on the significance of rs10183914, suggesting that their inclusion would be unlikely to influence the results. In this study only the original Fisher grade [30] was available; the updated modified Fisher grade [51] has been shown to be more predictive of outcome [52] and should be used in future studies when available.

In conclusion, in this study we identify and validate the *NFE2L2* SNP rs10183914 as associated with outcome in humans following aSAH and provide *in silico* functional evidence of the potential functional effect of this variant. This provides insight into the pathophysiological mechanisms of injury following aSAH and emphasizes the importance of oxidative stress. The data presented here further support NRF2 as a therapeutic target following aSAH and have implications for the management of other haemorrhagic stroke conditions.

## FUNDING INFORMATION

The GOSH study is funded by the Stroke Association. The study received funds from the Medical Research Council (MR/L01453X/1). Ben Gaastra is funded by the Royal College of Surgeons, Society of British Neurological Surgeons, Barrow Foundation UK and the Guarantors of Brain. We acknowledge the support from the Netherlands Cardiovascular Research Initiative: An initiative with support of the Dutch Heart Foundation, CVON2015‐08 ERASE. The Utrecht cohort has received funding from the European Research Council under the European Union's Horizon 2020 research and innovation programme (grant agreement No. 852173).

## CONFLICTS OF INTEREST

Diederik Bulters conceived and was chief investigator for the SFX‐01 after SAH (SAS) randomized controlled multicentre trial, sponsored by Evgen Pharma. Diederik Bulters and Ian Galea have consulted for Evgen Pharma and Bio Products Laboratory Limited, and have received research support from Bio Products Laboratory Limited.

## Supporting information


Table S1‐S4‐Figure S1

## Data Availability

Study data will be available from the authors subject to institutional agreements and ethical approvals.
