# Supplementary material for: Genetic variation in NFE2L2 is associated with outcome following aneurysmal subarachnoid haemorrhage
Source: Eur J Neurol. 2022 Oct 2;30(1):116–24. doi: 10.1111/ene.15571 (PMC10092511; doi:10.1111/ene.15571)
Supplement: Supplementary file 1 — Table S1‐S4‐Figure S1 [file ENE-30-116-s001.docx]

SUPPLEMENTAL MATERIAL

**Genetic variation in *NFE2L2* is associated with outcome following aneurysmal subarachnoid haemorrhage**

Ben Gaastra, Poppy Duncan, Mark K Bakker, Isabel C. Hostettler, Varinder S. Alg, Henry Houlden, Ynte M. Ruigrok, Ian Galea, Will Tapper, David Werring, Diederik Bulters

| SNP | Base pair | Minor allele | Number of samples | Odds ratio (95% CI) | P value |
| --- | --- | --- | --- | --- | --- |
| rs13035806 | 177227094 | A | 1035 | 1.33 (0.99-1.79) | 0.055 |
| rs2706110 | 177227434 | T | 1028 | 1.15 (0.91-1.46) | 0.243 |
| rs6726395 | 177238501 | A | 1036 | 1.22 (1.00-1.48) | 0.051 |
| rs10930781 | 177249904 | A | 1051 | 1.07 (0.75-1.50) | 0.722 |
| rs1806649 | 177253424 | T | 1040 | 1.18 (0.94-1.47) | 0.147 |
| rs2364723 | 177261818 | C | 1029 | 0.85 (0.69-1.05) | 0.138 |
| rs6706649 | 177265343 | T | 1031 | 1.06 (0.79-1.42) | 0.693 |
| rs35652124 | 177265345 | C | 1036 | 0.88 (0.72-1.09) | 0.237 |

Table S1. Multivariable logistic regression for non-significant tagged SNPs in GOSH cohort. Odds ratio reported with respect to the minor allele. Base pair reported in reference to hg38.

| SNP | Predictor | Odds ratio (95% CI) | P value |
| --- | --- | --- | --- |
| rs10183914 | SNP  Age  WFNS grade  1  2  3  4  5  Fisher grade  1  2  3  4  Treatment  Conservative  Endovascular  Surgical  Follow up  Sex  Female  Male | 1.27 (1.04-1.55)  1.02 (1.01-1.03)  Reference  1.56 (1.09-2.23)  2.54 (1.36-4.76)  3.78 (2.36-6.05)  4.32 (2.48-7.52)  0.55 (0.29-1.03)  0.54 (0.37-0.78)  0.66 (0.46-0.96)  Reference  0.68 (0.14-3.23)  0.71 (0.50-1.01)  Reference  0.99 (0.99-1.00)  0.76 (0.56-1.05)  Reference | 0.021  0.006  0.015  0.004  <0.001  <0.001  0.060  0.001  0.028  0.632  0.056  0.052  0.092 |
| rs6433657 | SNP  Age  WFNS grade  1  2  3  4  5  Fisher grade  1  2  3  4  Treatment  Conservative  Endovascular  Surgical  Follow up  Sex  Female  Male | 1.24 (1.02-1.50)  1.02 (1.00-1.03)  Reference  1.61 (1.13-2.31)  2.67 (1.42-5.03)  3.78 (2.35-6.09)  4.36 (2.51-7.59)  0.57 (0.30-1.07)  0.57 (0.39-0.83)  0.69 (0.47-0.99)  Reference  0.62 (0.13-2.96)  0.65 (0.46-0.93)  Reference  0.99 (0.99-1.00)  0.76 (0.56-1.04)  Reference | 0.034  0.008  0.009  0.002  <0.001  <0.001  0.079  0.003  0.044  0.550  0.017  0.071  0.086 |

Table S2. Multivariable logistic regression of significant SNPs for GOSH cohort reporting odds ratios for covariates included in model.

|  | Genetic variant and genotype | | | | | |
| --- | --- | --- | --- | --- | --- | --- |
|  | rs10183914 (n=1007) | | | rs64336578 (n=1002) | | |
|  | CC (n=451) | CT (n=418) | TT (n=138) | GG (n=327) | AG (n=455) | AA (n=220) |
| Age  Mean (range) | 53.5 (19-88) | 53.9 (22-92) | 53.2 (24-88) | 52.6 (19-88) | 54.8 (21-92) | 53.0 (22-88) |
| WFNS score  1  2  3  4  5 | 261 (58%)  101 (22%)  18 (4%)  40 (9%)  31 (7%) | 230 (55%)  94 (22%)  23 (6%)  42 (10%)  29 (7%) | 75 (54%)  30 (22%)  8 (6%)  17 (12%)  8 (6%) | 199 (61%)  69 (21%)  11 (3%)  26 (8%)  22 (7%) | 246 (54%)  105 (23%)  25 (5%)  44 (10%)  35 (8%) | 121 (55%)  49 (22%)  12 (5%)  27 (12%)  11 (5%) |
| Fisher grade  1  2  3  4 | 29 (6%)  135 (30%)  113 (25%)  174 (39%) | 40 (10%)  113 (27%)  91 (22%)  174 (42%) | 9 (7%)  51 (37%)  30 (22%)  48 (35%) | 18 (6%)  103 (31%)  85 (26%)  121 (37%) | 42 (9%)  119 (44%)  96 (21%)  198 (44%) | 17 (8%)  76 (35%)  50 (23%)  77 (35%) |
| Time to follow up (months)  Mean (range) | 25.3 (0-96) | 23.5 (0-96) | 25.2 (1-96) | 24.0 (0-95) | 24.8 (0-96) | 24.3 (0-92) |
| Treatment  Conservative  Endovascular  Surgical | 3 (1%)  367 (81%)  81 (18%) | 4 (1%)  330 (79%)  84 (20%) | 1 (1%)  105 (76%)  32 (23%) | 1 (0%)  255 (78%)  71 (22%) | 5 (1%)  368 (81%)  82 (18%) | 2 (1%)  179 (81%)  39 (18%) |
| Sex  Female  Male | 320 (71%)  131 (29%) | 297 (71%)  121 (29%) | 96 (70%)  42 (30%) | 222 (68%)  105 (32%) | 331 (73%)  124 (27%) | 153 (70%)  67 (30%) |
| Outcome  mRS 0  mRS 1  mRS 2  mRS 3  mRS 4  mRS 5  mRS 6  Good outcome  Poor outcome | 166 (37%)  160 (35%)  63 (14%)  26 (6%)  15 (3%)  5 (1%)  16 (4%)  326 (72%)  125 (28%) | 121 (29%)  154 (37%)  60 (14%)  40 (10%)  14 (3%)  9 (2%)  20 (5%)  275 (66%)  143 (34%) | 43 (31%)  44 (32%)  26 (19%)  16 (12%)  4 (3%)  1 (1%)  4 (3%)  87 (63%)  51 (37%) | 122 (37%)  117 (36%)  43 (13%)  19 (6%)  9 (3%)  4 (1%)  13 (4%)  239 (73%)  88 (27%) | 135 (30%)  167 (37%)  63 (14%)  39 (9%)  19 (4%)  9 (2%)  23 (5%)  302 (66%)  153 (34%) | 72 (33%)  70 (32%)  42 (19%)  23 (10%)  6 (3%)  2 (1%)  5 (2%)  142 (65%)  78 (35%) |

Table S3. Baseline covariates for significant SNPs in the GOSH cohort categorised by genotype.

| Haplotype | Frequency | Odds ratio | P value |
| --- | --- | --- | --- |
| GCCGGCCCCG | 0.292 | 0.889 | 0.281 |
| GCCGGCGCTG | 0.086 | 0.585 | 0.009* |
| GCTAGTGCTA | 0.216 | 1.160 | 0.198 |
| GCCGGCGCTA | 0.020 | 0.985 | 0.969 |
| GCCGGCGTTG | 0.127 | 1.070 | 0.642 |
| GTCAACGCTA | 0.066 | 0.901 | 0.617 |
| GCCAACGCTA | 0.020 | 1.390 | 0.353 |
| ATTAGCGCTA | 0.099 | 1.390 | 0.048* |
| ATTAGCGCTG | 0.012 | 1.280 | 0.551 |

Table S4. Multivariable logistic regression haplotype analysis for GOSH cohort. Greyed out bases represent the significant SNPs from the discovery analysis. * signifies < 0.05.

Figure S1. Histogram of time to follow up (months) in the GOSH cohort.
